# (F)utility of preoperative pulmonary function testing in pectus excavatum to assess severity

**DOI:** 10.1007/s00383-024-05675-3

**Published:** 2024-04-08

**Authors:** Gabriel C. Gonzalez, Alejandra M. Casar Berazaluce, Todd M. Jenkins, William D. Hardie, Karla E. Foster, Ryan A. Moore, Adam W. Powell, Victor F. Garcia, Rebeccah L. Brown

**Affiliations:** 1 College of Medicine, University of Cincinnati, Cincinnati, Ohio USA; 2 Division of Pediatric Surgery, Hasbro Children’s Hospital, Warren Alpert Medical School of Brown University, Providence, Rhode Island USA; 3 Department of Pediatric Surgery, Cincinnati Children’s Hospital Medical Center, 3333 Burnet Avenue, Cincinnati, Ohio USA; 4 Department of Pulmonary Medicine, Cincinnati Children’s Hospital Medical Center, Cincinnati, Ohio USA; 5 Cincinnati Children’s Hospital Medical Center Heart Institute, Cincinnati, Ohio USA

**Keywords:** Pectus excavatum, Pulmonary function test, Preoperative, Spirometry, Adolescent, Cardiopulmonary

## Abstract

**Purpose:**

The utility of pulmonary function testing (PFT) in pectus excavatum (PE) has been subject to debate. Although some evidence shows improvement from preoperative to postoperative values, the clinical significance is uncertain. A high failure-to-completion rate for operative PFT (48%) was identified in our large institutional cohort. With such a high non-completion rate, we questioned the overall utility of PFT in the preoperative assessment of PE and sought to evaluate if other measures of PE severity or cardiopulmonary function could explain this finding.

**Methods:**

Demographics, clinical findings, and results from cardiac MRI, PFT (spirometry and plethysmography), and cardiopulmonary exercise tests (CPET) were reviewed in 270 patients with PE evaluated preoperatively between 2015 and 2018. Regression modeling was used to measure associations between PFT completion and cardiopulmonary function.

**Results:**

There were no differences in demographics, symptoms, connective tissue disorders, or multiple indices of pectus severity and cardiac deformation in PFT completers versus non-completers. While regression analysis revealed higher RVEF, LVEF, and LVEF-Z scores, lower RV-ESV/BSA, LV-ESV/BSA, and LV-ESV/BSA-Z scores, and abnormal breathing reserve in PFT completers vs. non-completers, these findings were not consistent across continuous and binary analyses.

**Conclusions:**

We found that PFT completers were not significantly different from non-completers in most structural and functional measures of pectus deformity and cardiopulmonary function. Inability to complete PFT is not an indicator of pectus severity.

## Introduction

Pectus excavatum (PE) is a depression of the sternum into the thoracic cavity and is the most common congenital chest wall deformity [1–3]. While some patients exhibit signs of pectus excavatum at birth, many will not develop the deformity until early adolescence [4]. The severity of deformity and presence of symptoms such as chest pain and shortness of breath is variable amongst patients, and recent studies have suggested that the degree of compression has some association with the cardiopulmonary profile of the patient [5, 6]. The issue on whether the physiologic function improves with surgical repair of the deformity is controversial and a consensus in the literature has yet to be determined [6–12].

Pulmonary function testing (PFT) is a commonly used perioperative modality to assess the lung volumes and flows of these patients before and after repair [6, 13–20]. While routinely obtained at many centers, critics have suggested PFTs do not accurately represent exercise tolerance and their utility has been questioned [20, 21]. A recent study found very little correlation with cardiopulmonary testing including PFTs with patient-reported symptoms and concluded that routine PFT testing is generally not recommended in asymptomatic patients and should be reserved for symptomatic patients with the most severe depressions [21]. These tests are still routine at many centers across the country because insurance companies often require proof of impaired pulmonary function when assessing coverage for this patient population [21]. If preoperative PFTs lack clinical utility or consistency across this patient population, they could represent an extra cost and burden for these patients and our healthcare system.

In a cohort of pectus excavatum patients followed by our center from 2015 to 2018, we recorded that 48% of patients were either unable to perform PFTs or were only able to partially perform PFTs. With such a large incompletion rate, we have sought to investigate further the utility of PFTs at our center. It was not clear from our data whether the reason for inability to complete PFTs was due to a higher severity of disease or if there were other underlying contributing factors. This study sought to evaluate other structural or functional markers of pectus excavatum severity between patients who were able to perform PFTs and those who either partially performed or were unable to perform any acceptable PFTs and assess if any patterns existed between these groups.

## Materials and methods

This study used a database of 270 preoperative patients with the diagnosis of pectus excavatum at the Cincinnati Children’s Hospital Chest Wall Center from the years 2015 to 2018. For the purposes of the study, PFT completers were defined as those who were able to perform acceptable and repeatable spirometry and plethysmography, whereas PFT non-completers were designated as those who were only able to perform part of the test, or not at all according to the American Thoracic Society guidelines [22, 23]. These two groups were then retrospectively compared with results of cardiac MRI and cardiopulmonary exercise testing (CPET).

Spirometry and plethysmography were performed on one of three flow-sensing spirometers and whole-body boxes (Vyaire, Yorba Linda, CA) with calibration performed prior to testing daily also per American Thoracic Society (ATS) recommendations [22, 23]. Only pre-bronchodilator maneuvers were evaluated. The respiratory therapists supervising tests evaluated if each maneuver met all ATS criteria for acceptability and testing was discontinued once the patient produced efforts which met acceptable criteria that were also repeatable. These tests that fulfilled these ATS criteria and were reproducible were classified as acceptable and repeatable [22]. This is the group that we are describing as “PFT completers.” Spirometry which did not reach end of ATS test criteria but had satisfactory start and no artifacts with repeatable FEV1 were classified as usable, but not complete. Testing was discontinued if the subject was unable to produce any acceptable, usable, or repeatable efforts after 8 attempts. Patients in this group performed PFTs but were not able to fulfill the ATS test criteria or were considered usable we are describing as “PFT non-completers” [22, 23].

Cardiac MRI was performed, on a 1.5 Tesla scanner (Ingenia, Philips Healthcare; Best, Netherlands), to assess pectus severity, cardiac compression, and cardiac function. Cardiac functional imaging was performed using a standard retrospective ECG-gated, segmented steady-state free precession (SSFP) technique and included a short axis stack of cine SSFP images [24, 25]. The following pectus and cardiac deformation indices were measured: Haller index (HI), correction index (CI), depression index (DI), cardiac compression index (CCI), cardiac asymmetry index (CAI), and degree of sternal torsion (ST). These anatomic indices represent objective data obtained through cardiac MRI to assess the structural profile of the patients with pectus excavatum [26–31]. The following cardiac variables were collected: right ventricle (RV) and left ventricle (LV) ejection fraction (EF %), end-diastolic volume (EDV mL), end systolic volume (ESV mL), stroke volume (mL), mass (grams), and calculated values of body surface area (BSA) in. For binary analysis, we defined abnormal cardiac function as meeting one of the following criteria: RVEF < 50%, LVEF < 55%, and Z-scores < -2, as previously described in a study at our center assessing cardiopulmonary outcomes of pectus excavatum [6]. Z-score were obtained from volumetric and functional values from separate databases and abnormal RV and LV stroke volumes were defined according to referenced values based on age and sex [32, 33].

Exercise capacity was assessed with cardiopulmonary exercise testing (CPET) performed on the cycle ergometer using a ramping protocol. Continuous breath-by-breath gas analysis (Vmax Encore metabolic cart, VyaireMedical), forehead pulse oximetry, and 12-lead electrocardiogram tracings were recorded throughout the exercise [34]. Data collected included maximum oxygen uptake (VO_2_ peak) [% predicted], O_2_ pulse [% predicted], work [% predicted], and breathing reserve (BR) [%], and overall CPET interpretation [34]. For binary analysis, we defined abnormal CPET as peak VO_2_ < 80% predicted, O_2_ pulse < 80% predicted, work < 80% predicted, and breathing reserve (BR) < 12%, consistent with other studies assessing cardiopulmonary impact of pectus excavatum performed at our center [6, 19, 34, 35]. T-tests, Wilcoxon rank-sum tests, and chi-square tests were used to compare the after mentioned metrics by PFT completion status.

Linear and logistic regression modeling were then used to measure associations between PFT completion and cardiopulmonary function, adjusting for pectus and cardiac deformation indices, age, sex, race, and presence of the following symptoms: chest pain, shortness of breath, heart palpitations, and exercise intolerance. SAS v9.4 was used for all analyses and *p* < 0.05 was considered statistically significant. This study was conducted with approval from the Cincinnati Children’s Hospital Medical Center (CCHMC) Institutional Review Board (IRB) (IRB # 2018–3175).

## Results

**Fig. 1:**
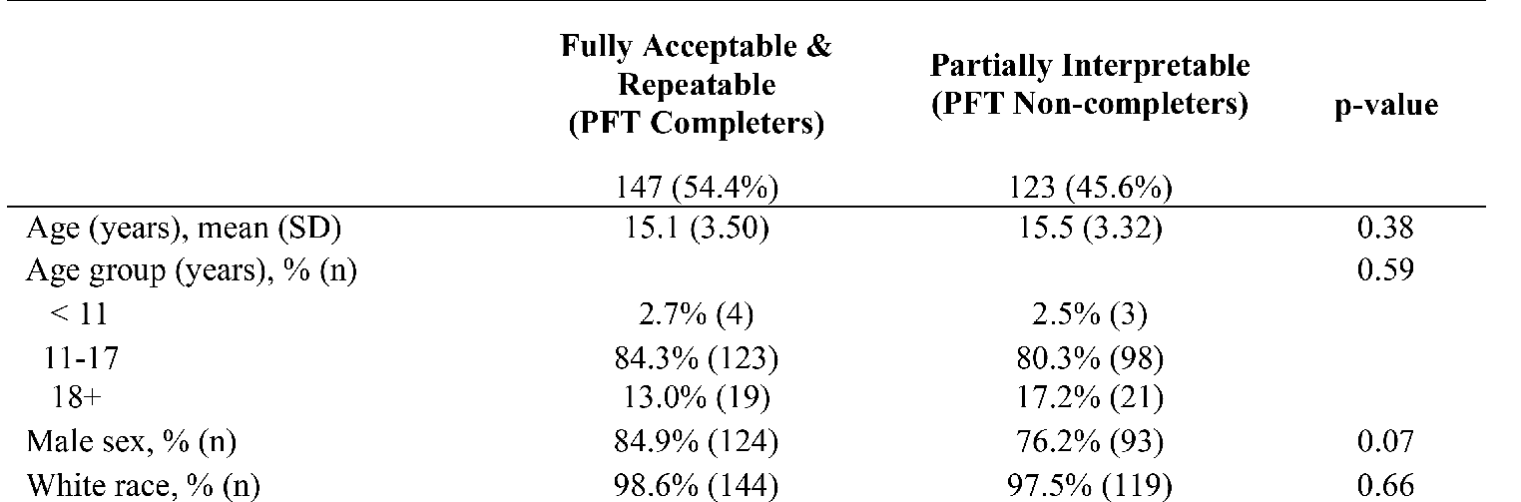
Demographic information of study participants comparing PFT completers vs. PFT non-completers

Of the 270 patients with pectus excavatum undergoing preoperative PFT, 54.4% of tests met fully acceptable and repeatable criteria (PFT completers), while the remainder (45.6%) of tests were considered unusable or patients were unable to perform the testing entirely (PFT non-completers). Further comparing these two groups, (see Fig. 1), the mean age of PFT completers was 15.1 years and the mean age of the non-completers was 15.5 years. The majority in both were male (84.9% vs. 76.2%) and almost all were white (98.6% vs. 97.5%).

**Fig. 2:**
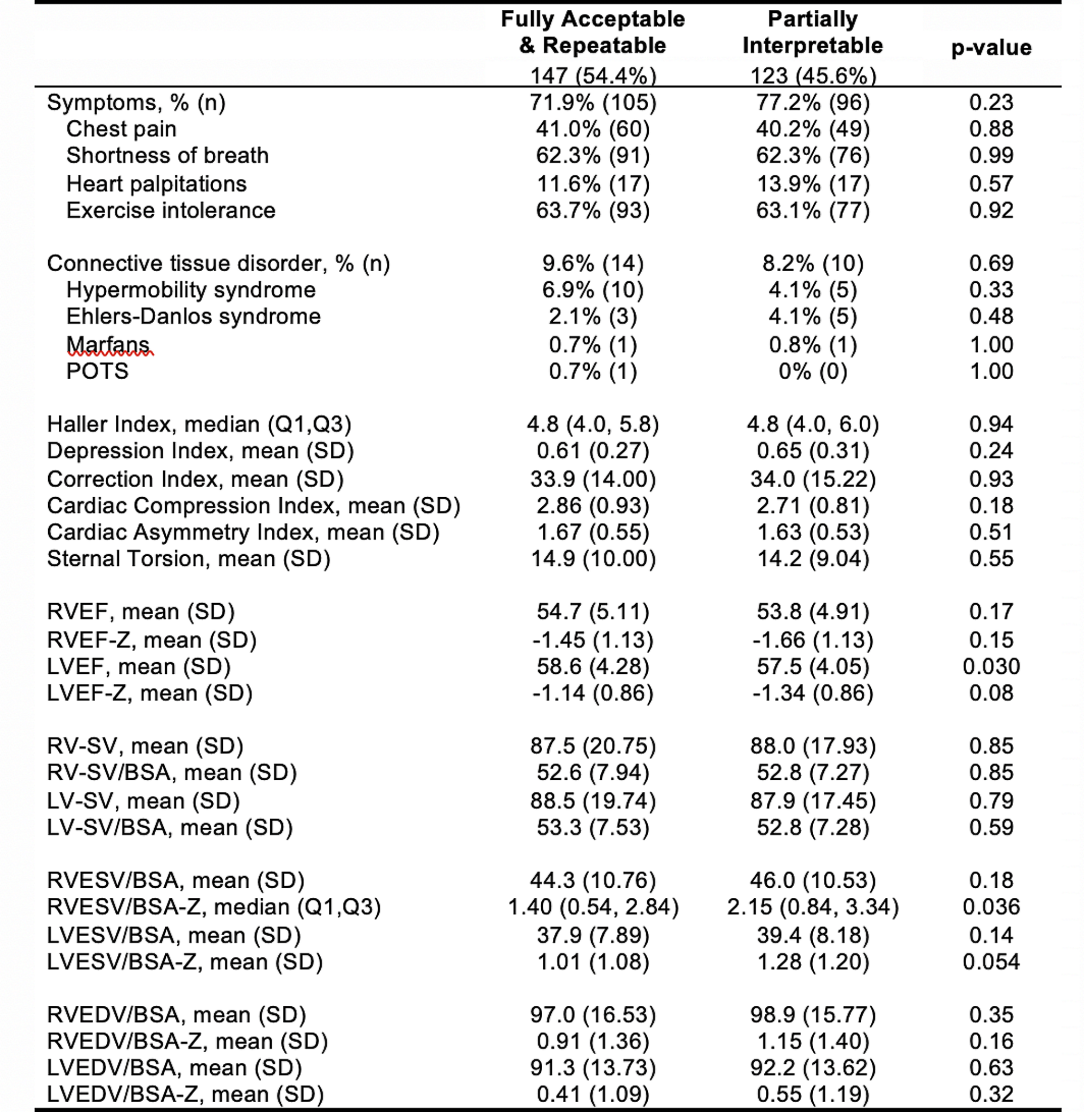
Reported symptoms, connective tissue disease status, and cardiac MRI findings for study participants

As shown in Fig. 2, there was no significant difference in patient-reported symptoms including chest pain, shortness of breath, heart palpitations, and exercise intolerance in PFT completers compared to PFT non-completers. There was also no difference in prevalence of connective tissue disorders between the two groups. Data from cardiac MRI showed no significant difference in the structural deformity indices including HI, CI, DI, CAI, CCI, and sternal torsion. Measures of cardiac function including RVEF, RV-SV, and LV-SV along with adjustments for body surface area were not found to be different between the two groups. There was a significant difference in mean LVEF (*p* = 0.03) and median RV-ESV/BSA-Z (*p* = 0.036).

**Fig. 3:**
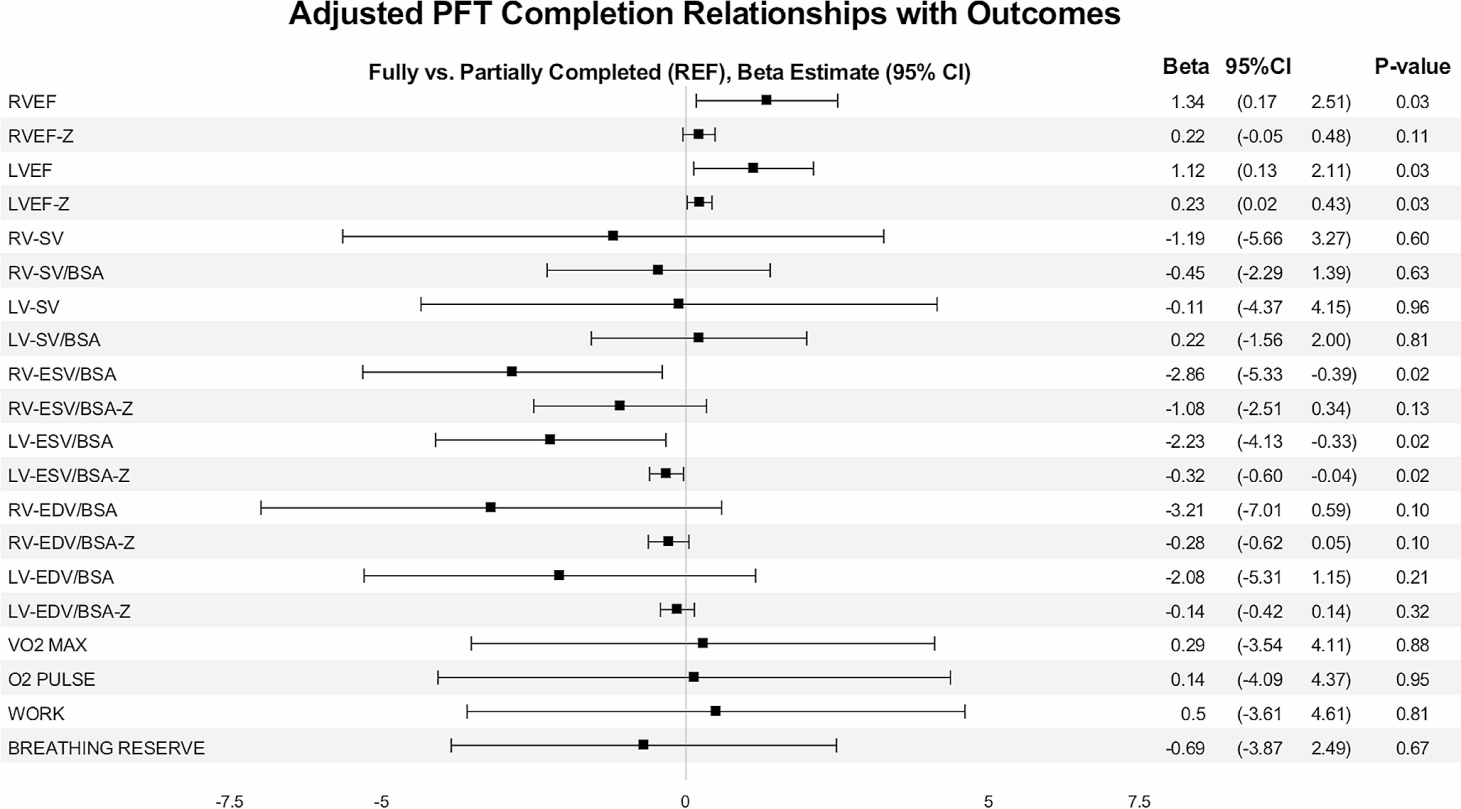
Continuous analysis of cardiac MRI and CPET data

**Fig. 4:**
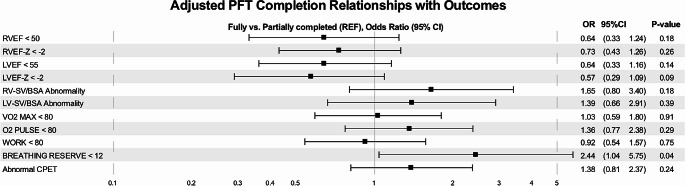
Binary analysis of cardiac MRI and CPET data

In adjusted continuous regression analysis (see Fig. 3), there were a few significant differences in measures of cardiac function between the two groups – including a higher RVEF (B estimate 1.34, *p* = 0.03), LVEF (B estimate 1.12, *p* = 0.03), and LVEF-Z (B estimate 0.23, *p* = 0.03) and a lower RV-ESV/BSA (B estimate − 2.86, *p* = 0.02), LV-ESV/BSA (B estimate − 2.23, *p* = 0.02), and LV-ESV/BSA-Z (B estimate − 0.32, *p* = 0.02) in PFT completers vs. non-completers. In adjusted binary regression analysis (see Fig. 4), the only significant finding was that PFT completers were more likely to have an abnormal breathing reserve < 12% (*p* = 0.04). There were no other significant differences in abnormal cardiac function or exercise capacity between the groups.

## Discussion

The use of preoperative PFTs is standard practice at many centers to assess pulmonary function before surgical repair of pectus excavatum [6, 13–20]. Previous studies evaluating pulmonary function before and after surgical repair and have found an association between a higher Haller Index (HI) and abnormal PFTs [36, 37]. In 2011, Lawson et al. reported that pectus patients with a HI > 7 are four times more likely to have reduced PFTs compared to the normal population and those with a HI < 4 [37]. However, despite this reduction, the preoperative FEV1, FVC, FRC, and TLC were generally still in the normal to low-normal range for most patients, even those with more severe HI [37].

It is estimated that PFTs are abnormal in about 20–25% of pectus patients [21, 37, 38]. In a large study that looked at respiratory function in 1342 primary school students, 35 of which had pectus excavatum, PFT results were normal in 87.9% of the controls versus 68.6% in those with pectus excavatum [39]. Our group recently reported abnormal PFTs in 23% of 281 patients undergoing preoperative testing for pectus excavatum [6]. Oftentimes, static PFTs obtained when the patient is at rest are entirely normal in pectus patients, and abnormalities are only identified during times of physiological stress such as exercise [39]. Unless concordant cardiopulmonary exercise testing is performed, the role of obtaining static PFTs at all has been appropriately questioned [20, 21].

Of the PFTs that are abnormal, it has been shown in the literature that pectus excavatum is generally associated with a restrictive pattern - theorized from the compression of the sternum on pulmonary structures during exhalation [20, 37]. More recently in 2016, 82 patients with pectus excavatum underwent preoperative PFT with a mixed distribution of 45% with a restrictive pattern (FVC < 80%), 35% with an obstructive pattern (FEV_1_ < 75%), and 25% with an indeterminate pattern [20]. The authors partially attribute this variability in PFT pattern to the median Haller Index in their population index which was 3.5 (range 2.8–5.2), milder than the patients described in other studies [20]. This was also researched at our center using this same cohort of pectus patients, and it was found that 10.7% of these patients had an obstructive pattern, 7.5% had non-specific ventilatory limitation (NSVL), and 7.5% had a restrictive pattern [40]. The obstructive and NSVL patterns in our study were comparable to the general population and the restrictive pattern was more commonly found in pectus patients [20, 40].

Considering the low percentage of abnormal preoperative PFTs and the lack of a consistent pattern in those that are abnormal, the actual utility of PFTs in the preoperative stage should be challenged. Our center has a considerably large non-completion rate for preoperative PFTs at 48%, and from our analysis it does not appear that there is a correlation between those who were able to fully perform versus those who either could not or were able to partially perform preoperative PFTs. We found a significant difference in RVEF (*p* = 0.03), LVEF (*p* = 0.03) and RVESV/BSA-Z (*p* = 0.036) but considering the lack of consistency among binary and continuous statistical analyses, these findings are likely not clinical meaningful. The cardiac stroke volumes are largely similar between both groups, so even with a slight difference in EF or ESV, there does not appear to be any further evidence that there is a difference in emptying the left or right ventricle between groups. A breathing reserve < 12% on CPET in PFT completers was the only variable significant on binary analysis, and due to the fact breathing reserve requires a reliable PFT and FEV1 to accurately assess, this finding is also not likely clinically significant. Our initial question was to explore if there was any pattern in those individuals who are completing the preoperative PFTs compared to those who are not, and our analysis concludes that there is not an obvious underlying factor.

Spirometry is an effort-dependent test and is described to be inherently difficult to perform, regardless of the presence of a thoracic wall deformity. Previous studies at our institution showed that for PFT performed for variable indications in patients age 4–17, only 74% of the tests were acceptable and repeatable on the initial attempt [41]. In this above-mentioned study, 85% of children older than 10 years old were able to perform acceptable and repeatable spirometry results, compared to 64% (7/11) of the patients identified as having pectus excavatum [41]. With this data and a larger identified patient population, only 52% of pectus patients were able to perform acceptable and repeatable spirometry results. While age could certainly have an impact, these spirometry tests appear to be difficult for adolescent-aged children in general, and there especially appears to be a lack of consistency of the ability of pectus patients to complete PFTs. To our knowledge, most studies assessing PFTs fail to mention if pulmonary function testing meets ATS standards. Our data highlights the importance of describing the quality of pulmonary function testing to ensure accurate reporting of lung function in pectus patients.

The utility of PFTs has recently been addressed by a recent study published by a team based at Montreal Children’s Hospital who analyzed 119 patients with pectus excavatum who underwent the Nuss procedure from 2004 to 2018 [21]. While they found an increased probability of abnormal PFT results in patients with HI greater than one standard deviation above the mean index (HI > 4.8), the prevalence of these results was below 20% [21]. They have since changed their practice to and now only obtain preoperative PFTs in symptomatic patients with HI > 4.8 [21].

In the United States, third-party insurers often require evidence of cardiopulmonary compromise before they will approve the Nuss procedure in patients with pectus excavatum [21]. This required documentation may represent a reason why preoperative PFTs are still obtained routinely across many large centers in the United States [21]. These tests are an added expense to the healthcare system and are an increased time burden for patients to complete.

Some limitations of our study include a limited sample size originating from a single institution. Our patient population was also largely white and male. A more diverse pool of patients could provide a more representative sample and increase the generalizability of our conclusions. Furthermore, our study does not provide specific data on why patients were not able to complete their PFTs, leaving it up to speculation. Having documented reasoning behind the 48% non-completion rate would have provided an additional variable to assess giving stronger insight on the characteristics of completers compared to non-completers.

## Conclusion

This study supports that patients able to perform acceptable and repeatable PFTs were not significantly different from those who could either partially perform or were unable to perform any acceptable PFTs in most structural parameters of pectus excavatum deformity and functional measures of performance. Therefore, the inability to perform a good quality PFT is not an indicator of severity in pectus excavatum or physiologic function measured by Cardiac MRI and CPET. This study did not evaluate rates of completion of postoperative PFT and its associated correlations with patient severity and cardiopulmonary status. Further investigation should be conducted to explore these alternate considerations.

## Data Availability

No datasets were generated or analysed during the current study.
